# Duplex ultrasound: A diagnostic tool for carotid stenosis management in type 2 diabetes mellitus

**DOI:** 10.4102/phcfm.v5i1.414

**Published:** 2013-07-24

**Authors:** Yogan Kisten, Pravesen Govender, Nadraj G. Naidoo, Dhiro Gihwala, Ferial Isaacs

**Affiliations:** 1 Department of Radiography, Cape Peninsula University of Technology, South Africa; 2 Faculty of Health & Wellness Sciences, Cape Peninsula University of Technology, South Africa; 3 Department of Surgery, Groote Schuur Hospital and University of Cape Town, South Africa

## Abstract

**Background:**

Diabetic patients are at increased risk of developing cardiac events and stroke, and prevention of diabetes mellitus is therefore desirable. Marked geographical and ethnic variation in the prevalence of diabetes caused by urbanisation, demographic and epidemiological transitions has rendered this one of the major non-communicable diseases in South Africa. Duplex ultrasound (DUS) plays an important role in primary health care in early detection of carotid atherosclerotic disease and the degree of carotid stenosis present. It is a reliable, cost-effective and non-invasive diagnostic tool. The purpose of this study was to determine the role of ultrasound in carotid stenosis management in type 2 diabetes mellitus (T2DM).

**Objectives:**

To determine the prevalence of carotid stenosis in a selected T2DM population using DUS and to correlate these findings with other predisposing atherosclerotic risk factors.

**Methods:**

The study setting was at an academic hospital in the Western Cape using carotid DUS reports of 103 diabetic subjects ≥ 35 years old. Predisposing risk factors were correlated with degree of carotid stenosis present. Data were analysed using the Fischer exact test, Chi-square and Student *t*-test.

**Results:**

Carotid DUS reports of 63 out of 103 T2DM patients revealed no evidence of a carotid stenosis, thereby lowering the risk profile. Forty patients were identified as having carotid stenosis; 22 symptomatic patients had a > 70% carotid stenosis which warranted surgical intervention. A greater prevalence of stenosis in the Caucasian group, in both the male (*p* = 0.0411) and female (*p* = 0.0458) cohorts, was noted. The overall trend suggested a relationship between T2DM and lifestyle, and a statistically significant relationship (*p* = 0.0063) between smoking and carotid stenosis was observed.

**Conclusion:**

T2DM and predisposing atherosclerotic risk factors significantly increased the possibility of carotid stenosis development.

## Abstrait

**L’échographie Duplex: Un outil de diagnostic de la prise en charge de la sténose carotidienne dans le diabète de type 2**

**Contexte:**

Les patients diabétiques courent un risque plus élevé de souffrir de crises cardiaques et d'attaques, la prévention du diabète de type 2 est donc souhaitable. Une variation géographique et ethnique marquée dans la prévalence du diabète causée par l'urbanisation et les transitions démographiques et épidémiologiques a fait du diabète l'une des principales maladies non transmissibles en Afrique du Sud. L’échographie Duplex (ED) joue un rôle important dans les soins de santé primaires en matière de détection précoce de l'athérosclérose carotidienne et le degré de sténose carotidienne présente. Il s'agit d'un outil de diagnostic fiable, économique et non invasif. L'objet de cette étude consistait à déterminer le rôle de l’échographie dans la prise en charge de la sténose carotidienne dans le diabète de type 2 (DT2).

**Objectifs:**

Déterminer la prévalence de la sténose carotidienne dans une population souffrant de DT2 sélectionnée en utilisant l'ED et corréler ces conclusions à d'autres facteurs de risque prédisposant à l'athérosclérose.

**Méthodes:**

Le cadre de l’étude consistait en un hôpital universitaire dans le Cap occidental et incluait l'utilisation des rapports de l'ED de 103 sujets diabétiques âgés de plus de 35 ans. Les facteurs de risque de prédisposition étaient corrélés au degré de sténose carotidienne présente. Les données ont été analysées en utilisant la méthode exacte de Fisher, le test du chi carré et le test t de Student.

**Résultats:**

Les rapports de l'ED de la carotide de 63 des 103 patients souffrant de DT2 n'ont révélé aucune preuve de sténose carotidienne, diminuant ainsi le profil de risque. Quarante patients ont été identifiés comme présentant une sténose carotidienne; 22 patients symptomatiques présentaient une sténose carotidienne > 70% qui nécessitait une intervention chirurgicale. Une prévalence de la sténose plus importante dans le groupe caucasien, à la fois chez les cohortes d'hommes (*p* = 0.0411) et de femmes (*p* = 0.0458) a été notée. La tendance générale indiquait une relation entre le DT2 et le mode de vie, et une relation statistiquement significative (*p* = 0.0063) entre le fait de fumer et la sténose carotidienne a été notée.

**Conclusion:**

Le DT2 et les facteurs de risque prédisposant à l'athérosclérose augmentaient significativement la probabilité de développer une sténose carotidienne.

## Introduction

Early identification of patients at high risk of cardiac events and stroke is known to improve preventive and clinical care.^1^ Type 2 diabetes mellitus (T2DM) is reported as the second most common risk factor for stroke in rural South Africans.^2^ There is marked geographical and ethnic variation in prevalence of diabetes caused by urbanisation and demographic and epidemiological transitions that have rendered this one of the major non-communicable diseases in South Africa.^3, 4, 5, 6^

Cardiovascular disease (CVD) is the leading cause of morbidity and mortality in patients with diabetes, who also have twice the risk of stroke or heart disease and a greater prevalence of atherosclerosis than patients who are not diabetic.^7, 8^ Many steps have been taken to reduce this risk by determining best methods for controlling the disease and preventing its complications.^7^ Stroke can be prevented if detected early enough.

Ultrasound imaging plays an important role in early detection of carotid atherosclerotic disease since it is able to quantify plaques formed and estimate the degree of carotid stenosis present. The technique allows determination of the severity of carotid stenosis by evaluating the extent of atherosclerotic changes and echo patterns within the vessel, using real-time B-mode ultrasound coupled with colour and spectral Doppler-imaging techniques (Duplex ultrasound or DUS). In general DUS is regarded as reliable for delineating atherosclerotic plaques with or without calcification.^9^ It is also useful in determining the severity of obstruction, the intima and media thickness, and the anatomical site of atherosclerotic involvement.^9^

Carotid bloodflow velocities can be analysed with the aid of Doppler waveform analysis, which further assists in diagnosis.^9^ Another advantage of carotid DUS is that it is non-invasive and more readily accepted by the patient.

Subjects with diabetes mellitus are more susceptible to macrovascular disease manifesting as coronary artery disease, stroke/cerebrovascular accident and peripheral arterial disease compared to non-diabetic subjects.^10^ It has also been determined that the increased frequency of dyslipidaemia, hyperglycaemia, obesity, hypertension and associated nephropathy may contribute to accelerated atherogenesis in diabetic subjects.^11^

Diabetes mellitus is considered to be a major risk factor for ischaemic stroke. Diabetic patients with predisposing risk factors have a greater incidence of stroke compared to non-diabetic subjects, but consensus is yet to be reached on optimal screening methods for cerebrovascular complications.^10^ Conventional digital subtraction angiography is currently considered to be inappropriate as a first-line investigation for diagnosing carotid stenosis, since it is an invasive and radioactive technique. Computed tomography angiography and magnetic resonance angiography provide more reliable anatomical resolution but are not cost-effective.

Carotid DUS is operator dependent; it requires trained vascular sonographers^12, 13^ in specialised vascular clinics and referral centres. However, at primary health care (PHC) level a basic, less expensive, portable ultrasound machine with appropriate carotid settings can be used to recognise patterns and measure and identify carotid wall thicknesses and plaques of individuals at risk of Cardiovascular disease (CVD) and stroke. This tool supplemented by clinical assessment allows the PHC professional to ‘flag’ high-risk individuals requiring referral and appropriate management at specialised centres.

Carotid artery ultrasound scanning therefore has potential to be the initial screening tool of choice in patients with carotid artery disease due to its safety, easy availability and cost-effectiveness. The efficacy of this non-invasive test is also a recommendation for PHC ultrasound in emergency medicine, general abdominal, obstetrics and gynaecology patients, to name a few.^1^

Atherosclerotic carotid artery disease is reported to be an independent predictor of cerebrovascular events.^14^ A combination of hypertension, increased total blood cholesterol level, poor lifestyle and diabetes mellitus is contributory to stroke and CVD. Currently in South Africa symptomatic patients (transient ischaemic attack or minor stroke) in public hospitals receive surgical treatment if the carotid artery stenosis is > 70%, as recommended by current guidelines.^15, 16, 17^ However, it has been suggested that the severity of atherosclerosis can be reduced if plaque development is detected as early as possible.^14, 15, 16, 17, 18, 19, 20, 21^ As such, ultrasound can contribute significantly in the early detection, surveillance and management of carotid artery disease.^14, 15, 16, 17, 18, 19, 20, 21, 22^

The degree of carotid stenosis was documented in available carotid DUS reports of patients with T2DM at Groote Schuur Hospital. This was used to determine prevalence of stenosis present in this sample population using mean age, smoking, ethnicity, body mass index (BMI) and gender as discriminating risk factors (many of which are modifiable). A problem in the South African setting is that patients present to specialised referral centres when their condition is already severe. Preventative strategies may well include ultrasound screening at PHC level.

Pattern recognition using handheld portable ultrasound machines to screen for obvious pathology is useful not only in cardiovascular imaging but also in general medicine. Basic ultrasound imaging as part of the medical curriculum may be beneficial in familiarising PHC professionals with this technology.

## Setting

A retrospective analysis was carried out at a dedicated vascular laboratory run by the vascular surgery unit at Groote Schuur Hospital, a tertiary academic hospital in Cape Town, South Africa.

## Significance of work

Ultrasound is a reliable, cost-effective and safe imaging modality used as an alternative to other diagnostic tools where scientific evidence supports its appropriateness. Sound wave technology continues to evolve, and when used in telemedicine it further contributes to PHC. However, technology transfer of ultrasound to rural parts of middle- and low-income countries like South Africa is not used to its full potential. Lack of human resources and infrastructure are major limitations in implementing preventative medical strategies.

Preventative strategies include early detection, lifestyle modification and appropriate management to reduce ill health. Physicians can only assess an individual's risk indirectly based on various risk factors. The possibility of directly depicting carotid atherosclerosis and the degree of stenosis present adds more value to an individual's risk assessment for CVD and stroke, for early intervention where necessary.

A relationship is reported to coexist between atherosclerosis, diabetes mellitus, CVD and stroke, which therefore complements the significance of responsible use of carotid ultrasound imaging as a diagnostic tool in management of patients with T2DM.

## Ethical considerations

Ethical approval was granted by the Cape Peninsula University of Technology Research Ethics Committee. Special permission was also granted by the department head of vascular surgery at Groote Schuur Hospital for use of subject ultrasound folders for the study. Strict ethical guidelines were adhered to.

## Methodology

Ultrasound reports of 200 patients were acquired from the medical record archive for the study period January–April 2008. Ninety seven patients were not diabetic and were subsequently excluded, whilst 103 patients were eligible for the study. The carotid DUS reports and patient clinical folders were reviewed. The data sheet used for analysis included age, gender, smoking, ethnicity, number of ultrasound scans done, BMI, degree of stenosis present, surgical intervention if required, and other medical conditions (hypertension and elevated total blood cholesterol level) if present. Using this data sheet the prevalence of carotid artery disease in this selected T2DM population was calculated.

## Results

A total of 103 ultrasound reports for T2DM patients was used in the study; 52 (50.4%) were for male patients and 51 (49.6%) for female patients****. Age range for male patients was 38–80 years (mean 60.3 years) and for females 38–92 years (mean 61.6 years), with no statistically significant difference (*p* = 0.5398) between the two in respect of age.

Sixty three of the patients were found to have a normal carotid DUS, thus lowering their risk profile. A total of 40 subjects in this selected population was found to have a carotid stenosis on DUS imaging. In 22 (55%) of the 40 subjects the stenosis was > 70%; these individuals were symptomatic and subsequently had carotid intervention. The other 18 subjects had a stenosis of < 70% and were treated medically, but had to avail themselves for serial follow-up ultrasound scans.

Of the 40 subjects with some form of a stenosis, only 3 were non-smokers and 37 (92.5%) were smokers. Of the 63 subjects without stenosis, 42 (66.6%) were smokers. From this it is concluded that there was a statistically significant relationship (*p* = 0.0063) between smoking and stenosis, as depicted in Table 1.

**TABLE 1:** Relationship between carotid stenosis and smoking.*

Predisposing factor	Stenosis	No stenosis	Total
Smoking	37	42	79
Non-smoking	3	21	24
	40	63	103

* *p* = 0.0063.

The data shown in Table 2 are uniquely classified according to the South African context of ethnic descent. In this study 39 subjects were Caucasians (of European decent), and the remaining 64 subjects had roots in either Asia or Africa. Of those of European decent, 12 of the 20 male and 10 of the 19 female subjects had a carotid stenosis present. Of the male subjects in the other ethnic groups, 10 of the 22 had a carotid stenosis whilst in females the figure was 8 of the 24. Statistically a greater proportion of males of European descent had a carotid stenosis (*p* = 0.0411) compared to males in the other groups (0.45). Similarly, females of European descent were more likely to have a carotid stenosis compared to females in the other groups.

**TABLE 2:** Relationship between South African ethnic groups and carotid stenosis.

Ethnicity	Male		Female		Total
	
	Stenosis	No stenosis	Stenosis	No stenosis	
Mixed ancestry	7	14	6	15	42
Asian descent	2	3	1	4	10
African descent	1	5	1	5	12
European descent	12	8	10	9	39

**Total**	**22**	**30**	**18**	**33**	**103**

Males *p =* 0.0411; females *p =* 0.0458.

**FIGURE 1:**
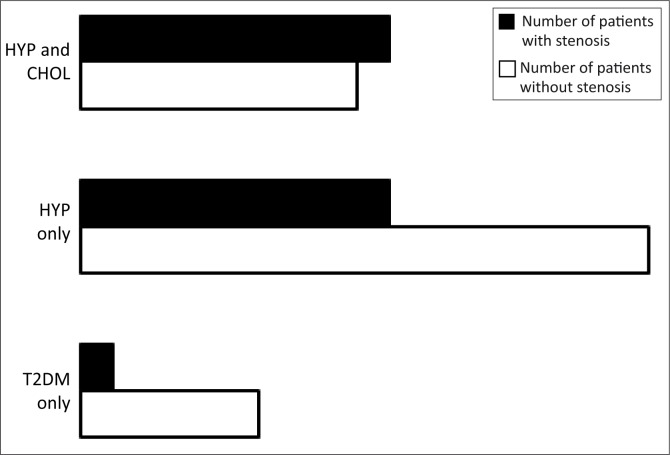
Association between hypertension and cholesterol (HYP&CHOL), hypertension only (HYP ONLY) and T2DM only and carotid stenosis.

Of those with hypertension and increased blood cholesterol levels, 19 had a carotid stenosis whilst 21 did not. Of those with hypertension and diabetes, a minority of 19 out of 54 and 2 out of 13 respectively had a carotid stenosis, as shown in Figure 1.

**FIGURE 2:**
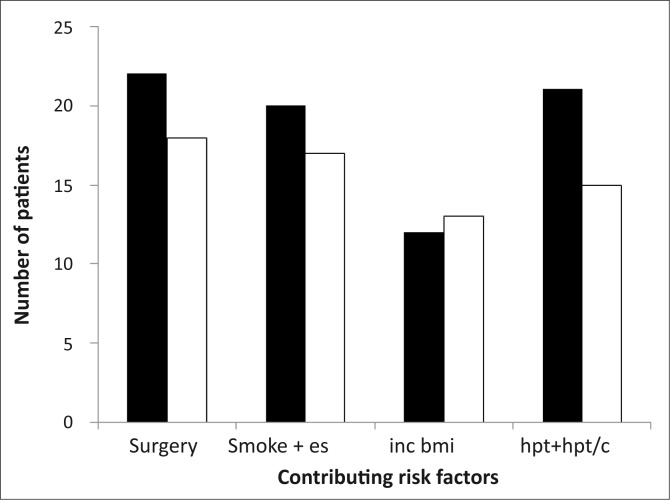
Possible risk factors (Smoking [smoke +es], increased BMI [inc bmi], hypertension and/or increased total blood cholesterol [hpt + hpt/c]) leading to surgery in subjects with carotid stenosis.

Of the 40 subjects that had stenosis, 22 were diagnosed sonographically as having a > 70% carotid stenosis and managed by surgical intervention (21 carotid endarterectomy and 1 carotid angioplasty and stenting). As shown in Figure 2, of the 22 subjects that required surgery 20 (*n* = 22) were smokers, 12 (*n* = 22) had an increased BMI and 20 (*n* = 22) had hypertension only or hypertension and increased blood cholesterol levels.

This study indicates that T2DM together with other risk factors increases the possibility of carotid stenosis compared to T2DM alone. Predictors of carotid stenosis included a combination of the subjects’ age, gender, smoking, ethnicity, BMI, degree of stenosis and other medical conditions such as hypertension and elevated total blood cholesterol level. These results are supported by previous published literature on risk factors associated with carotid stenosis.^2, 9, 12, 15, 16, 17, 18, 19, 20, 21^ These results also reflect a small sample collected over a four-month period, and are therefore only an analysis of the trend or patterns developed in T2DM subjects sonographically diagnosed with a stenosis. Severe atherosclerosis with a carotid stenosis of > 70% in this select group of patients with T2DM and other risk factors was considered to warrant surgery, which is usually done to treat or prevent stroke.^15, 16, 17, 18, 19, 20, 21, 21^

Of the 103 subjects diagnosed with T2DM included in the study, 40 had a carotid stenosis. The ultrasound findings played a role in excluding 63 of the subjects without a stenosis, hence lowering their risk profile and assisting in appropriate management and care. Twenty two of the 40 subjects (55%) had a stenosis of > 70%, which was detected by ultrasound and managed surgically. Of these 22 subjects that required surgery, all had other associated risk factors. There was a statistically significant relationship (*p* = 0.0063) between smoking and carotid stenosis (Table 1).

Fifty five per cent (*n* = 22) also had an increased BMI. The findings demonstrate a pattern, showing that lifestyle and other environmental factors contribute towards extracranial carotid disease development leading to surgical intervention. What was also noted in this study is that 18 (45%) had a stenosis < 70%; they did not have surgery but required follow-up ultrasound scans and optimum medical treatment (OMT). This demonstrates the useful role of ultrasound in identifying precisely those patients that require surgery and OMT, and those that only require OMT. The study verifies that increased frequency of dyslipidaemia, hyperglycaemia, obesity, hypertension and associated nephropathy may contribute to accelerated atherogenesis in diabetic patients.^11^

In terms of social groups, those of ‘European descent’ demonstrated the highest incidence of stenosis. In the context of South African society this group is subject to a more Western lifestyle that predisposes them to greater risk of atherosclerotic disease. This might indeed be exacerbated by hereditary factors subjecting them to this disposition. Higher carotid stenosis is also associated with urban living.^13^ Cultural and lifestyle changes due to urbanisation also contribute to development of T2DM, hypertension, increased blood cholesterol levels, smoking and increased BMI.

Apart from the above risks, age was another factor linked with a high prevalence of carotid stenosis and the likelihood of surgery. The average age for those in the study (*n* = 103) was 61 years, the minimum age being 38 years and the maximum 92 years. The youngest subject that had surgery was 52 years of age and the oldest was 78. This study demonstrates that older subjects (≥ 52 years) are at high risk of a carotid stenosis > 70%. It is exceptionally rare to document a young patient < 35 years having a carotid stenosis > 70% requiring surgical intervention in clinical practice.

## Discussion

Whilst the small sample size is a limitation; these results are important in establishing a trend for larger studies, and this study emphasises early detection of carotid artery disease using carotid artery ultrasound techniques. Spectral Doppler and B-mode imaging of carotid artery intima-media thickness measurements are recommended for further studies in order to identify atherosclerotic changes in subjects at high risk of stroke and CVD early.^17^ This will be of great value in PHC preventive medicine.

Ultrasound therefore has an important role to play in the management of subjects with T2DM, hypertension and increased total blood cholesterol levels. This in turn can be used as a screening tool for early detection of stroke and CVD. As a prevention campaign, awareness programmes of lifestyle changes in controlling the effects of T2DM, obesity, hypertension and blood cholesterol levels should be emphasised.^17^

Three-dimensional imaging^22^ has emerged as an additional ultrasound tool to calculate plaque volume for precise planning of surgical interventions. Future studies of three-dimensional ultrasound volume measurements of carotid arteries are recommended.

## Conclusion

This study found a high prevalence of carotid artery stenosis in T2DM South Africans of European descent, although other contributory risk factors need to be considered. This study was in a selected high-risk group from a specialised referral vascular clinic, and hence does not reflect the total Western Cape regional population. Results of this study correlate with supported literature that ultrasonography has a significant role to play in monitoring subjects with T2DM and associated risk factors.

If vascular disease is identified and treated early at PHC level, there would be less need for intervention at tertiary referral centres. Ultrasound imaging is cost-effective, non-invasive and easily accepted by the patient, and hence a valuable diagnostic tool in PHC. Ultrasound can also provide functional and anatomical information about vascular changes like carotid artery wall thickness, plaque morphology and stenosis.

With advancement in technology the resolution of images provided by ultrasound is improving exponentially, which bodes well for rapid, efficient and enhanced sensitivity for medical application at both tertiary and PHC level.
